# Influence of Nb on the Microstructure and Fracture Toughness of (Zr_0.76_Fe_0.24_)_100−x_Nb_x_ Nano-Eutectic Composites

**DOI:** 10.3390/ma11010113

**Published:** 2018-01-11

**Authors:** Tapabrata Maity, Anushree Dutta, Parijat Pallab Jana, Konda Gokuldoss Prashanth, Jürgen Eckert, Jayanta Das

**Affiliations:** 1 Department of Materials and Metallurgical Engineering, Indian Institute of Technology Kharagpur, Kharagpur 721302, India; maity.tapabrata@gmail.com or tapabrata.maity@unileoben.ac.at (T.M.); anushree032003@yahoo.co.in (A.D.); parijat.pallab@iitkgp.ac.in (P.P.J.); j.das@metal.iitkgp.ernet.in (J.D.); 2 Department of Materials Physics, Montanuniversitat Leoben, Jahnstrasse 12, 8700 Leoben, Austria; juergen.eckert@oeaw.ac.at or juergen.eckert@unileoben.ac.at; 3 Department of Manufacturing and Civil Engineering, Norwegian University of Science and Technology, Teknologivegen 22, 2815 Gjøvik, Norway; 4 Erich Schmid Institute of Materials Science, Austrian Academy of Sciences, Jahnstraße 12, A-8700 Leoben, Austria

**Keywords:** eutectic alloys, microstructure, mechanical properties, indentation fracture toughness, electron microscopy

## Abstract

The present study demonstrates the evolution of eutectic microstructure in arc-melted (Zr_0.76_Fe_0.24_)_100−x_Nb_x_ (0 ≤ x ≤ 10 atom %) composites containing α-Zr//FeZr_2_ nano-lamellae phases along with pro-eutectic Zr-rich intermetallic phase. The effects of Nb addition on the microstructural evolution and mechanical properties under compression, bulk hardness, elastic modulus, and indentation fracture toughness (IFT) were investigated. The Zr–Fe–(Nb) eutectic composites (ECs) exhibited excellent fracture strength up to ~1800 MPa. Microstructural characterization revealed that the addition of Nb promotes the formation of intermetallic Zr_54_Fe_37_Nb_9_. The IFT (*K_IC_*) increases from 3.0 ± 0.5 MPa√m (x = 0) to 4.7 ± 1.0 MPa√m (x = 2) at 49 N, which even further increases from 5.1 ± 0.5 MPa√m (x = 0) and up to 5.9 ± 1.0 MPa√m (x = 2) at higher loads. The results suggest that mutual interaction between nano-lamellar α-Zr//FeZr_2_ phases is responsible for enhanced fracture resistance and high fracture strength.

## 1. Introduction

Eutectic composites (ECs) are extensively studied because their properties are superior to those of monolithic single-phase alloys and single crystals. They have good chemical compatibility and exhibit high strength in ambient atmospheres and at elevated temperatures [1,2]. ECs have a low melting temperature, high castability, a controllable microstructure, and widespread engineering applications. Recently, high strength (2 GPa) and a high plasticity of 20–25% was achieved in Ti–Fe-, Ni–Zr-based lamellar ultrafine eutectic composites (UECs) by tuning the composition of bulk metallic glass (BMG) to synthesize a bulk specimen via arc melting and solidifying at low cooling rates [2,3,4,5,6,7,8,9,10,11,12,13,14,15]. In such cases, a high degree of constitutional super-cooling restricts the growth of eutectic phases within the nanometer scale towards the synthesis of nano-lamellar eutectic composites (NECs) [2,3,4,5,6,7,8,9,10,11,12,13,14,15]. The microstructural features of lamellar composites are crucial for tuning the deformation behavior of ECs. Several NECs, such as Ti–Fe–(Sn) [2,3,4,5,6,7], Ni–Zr–(Al) [8,9,10], Al–Cu–(Sn) [11], Al–Cu–(Al) [12,13], Fe–Nb–(Al/Mn/Ni) [14], Fe–Si–Ti [15], and Fe–Nb–(Al) [15], have been reported to have outstanding fracture strength (>2 GPa) along with large plastic strain (>15% at RT) [2,3,4,5,6,7,8,9,10,11,12,13,14,15,16]. It has been suggested that enhanced mechanical properties have been obtained through impingement of the propagating shear bands (SBs) and through the impingement of the movement of dislocations along with the pile-up of dislocations at the nano-/ultrafine lamellae interface, which promotes a strong work hardening behavior [14,15,16,17]. The lamellar eutectic provides nucleation of a large number of SBs, which accommodate the applied shear strain at the lamellae interfaces, eutectic colonies and slip band transfer. All the above said mechanisms are responsible for the significant tensile ductility of the eutectic microstructure [8,9,10,11,12,13,14,15,16,17].

Considering a critical survey of the current state of understanding, further exploration in the broad field of the mechanical properties of NECs, particularly in the context of processing, as well as the structure–property relationship seem to be essential. On the other hand, Zr-based alloys possess high-temperature properties, good mechanical and radiation damage resistance, low thermal neutron capture cross section, and excellent corrosion resistance, which makes them useful in nuclear power plant applications and chemical processing plants [17,18,19]. Zr–Fe eutectic composites are very attractive because of their high abundance of natural resources and corrosion-resistant properties [17,18,19]. However, very few studies have been reported on the structure–property relationship and phase formation, the deformation mechanism, and the fracture behavior of the Zr-base lamellar eutectic composites [20,21]. The present work attempts to reveal the microscopic deformation behavior of the Zr–Fe-based NECs. The effect of Nb addition to eutectic Zr_76_Fe_24_ on the compressive mechanical properties and the deformation behavior have been evaluated using the indentation fracture toughness method at various loads. 

## 2. Materials and Methods

A series of (Zr_0.76_Fe_0.24_)_100−x_Nb_x_ (x = 0, 2, 4, 6, and 10 atom %) alloy ingots were prepared by arc-melting process in an Ar atmosphere. The arc-melted ingots (AMIs) were re-melted repeatedly at least three times for homogenization. Parallelepiped specimens were cut from the AMIs using electro-discharge machining (EDM). The constituent phases and their structure were identified using a Philips PW3373 PANalytical high-resolution X-ray diffraction unit (XRD, Cu-K_α_ radiation, Philips, Kassel, Germany). The Vickers macro-hardness (*H*) of the specimens were measured using Leco LV-700, USA Vickers hardness tester (LECO, Saint Joseph, MO, USA) according to ASTM E-384 standard for a dwell time of 15 s. Indentations at each test load of *P_max_* = 49 N up to *P_max_* = 294 N were conducted for an average hardness *H* [4,8]. The measurement of IFT is sensitive to surface preparation; therefore, the samples were polished carefully to eliminate the residual stress zones on the specimens’ surface. The IFT values denoted as *K_IC_* was measured on highly polished specimen surfaces, which were free from any pre-cracks, using a Vickers diamond pyramid indenter at *P_max_* values of 49 to 294 N and the Vickers hardness tester mentioned above. *K_IC_* deals with the critical value of stress intensity factor *K_I_* in crack opening mode when fracture initiates and an unstable crack propagates. The *K_IC_* values of Zr–Fe–(Nb) eutectic composites alloys were calculated using an equation proposed by Niihara et al. [22,23]:

(1) $$K_{IC}=0.018(E^{0.4})\left(H^{0.6}\right)\left(l^{0.5}\right)\left(a\right)$$

(2) $$K_{IC}=0.0123(E^{0.4})\left(H^{0.1}\right){\left(\frac{P}{l}\right)}^{0.5}$$

where *H* is the macrohardness in GPa, *E* is Young’s modulus in GPa, *P* is the applied indentation load (*P_max_*), *a* is half of the indentation diagonal length, and *l* is the Palmqvist crack length, i.e., the extent of the cracks that emerge from the edges of the indentations only. A Leica DM 2500M optical microscope (OM) and a SUPRA 40 field emission scanning electron microscope (FESEM, Carl Zeiss SMT AG, Oberkochen, Germany) equipped with an Oxford ISIS300 energy dispersive X-ray spectrometer (EDS) (Oxford Instruments plc, Oxfordshire, UK) were used to investigate the geometry of the indentation impressions, the median, and Palmqvist crack lengths. The volume fraction of the phases were calculated from the microscopy images. At least five images were used to obtain average values. Compression tests (CTs) were performed at room temperature using a TINIUS Olsen H50KS universal testing machine (TINIUS Olsen, Kolkata, India) at an initial strain rate of 8 × 10^−4^ s^−1^. A detailed microstructural investigation was performed using a JEOL JEM-2100 transmission electron microscope (TEM) (JEOL, Tokyo, Japan). Thin TEM samples were prepared by mechanical polishing followed by ion-beam milling in liquid N_2_ using Gatan PIPS691 precision ion polishing system. A 35 DLP Olympus Panametric ultrasonic pulser receiver was used to measure the elastic properties.

## 3. Results and Discussion

### 3.1. X-ray Diffraction and Phase Analysis of As-Solidified Composites 

**Figure 1:**
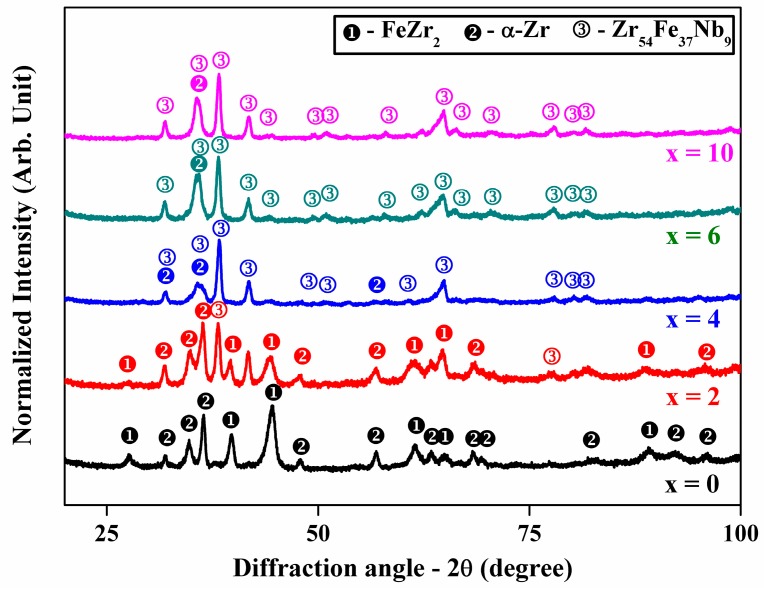
XRD patterns of (Zr_0.76_Fe_0.24_)_100−x_Nb_x_ (0 ≤ x ≤ 10 atom %) showing the presence of both α-Zr and FeZr_2_ peaks in samples with x < 3 and the presence of α-Zr and Zr_54_Fe_37_Nb_9_ phases in samples with x > 3.

Figure 1 shows the XRD patterns for the Zr–Fe–(Nb) with varying Nb content up to x = 10 atom %. The XRD pattern of x = 0 shows sharp diffraction peaks of hcp α-Zr, bcc FeZr_2_; however, additional reflections of intermetallic Zr_54_Fe_37_Nb_9_ (JCPDS #00-046-1095) has been observed from x = 2 [24]. It has been observed that the peak intensity of FeZr_2_ phase gradually decreases with the addition of Nb content, whereas the peak intensities of intermetallic Zr_54_Fe_37_Nb_9_ phase increases with the increase in the amount of Nb. This indicates that the phase fraction of intermetallic Zr_54_Fe_37_Nb_9_ is higher in the alloys with higher Nb content. Therefore, it may be concluded that the addition of Nb assists in the formation and stabilization of the intermetallic phase Zr_54_Fe_37_Nb_9_.

### 3.2. Microstructural Characterization of the As-Solidified Composites 

**Figure 2:**
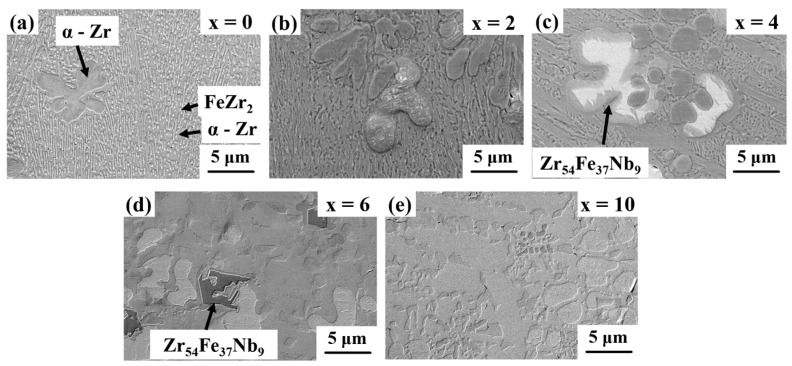
Scanning electron microscopy images of (**a**) x = 0; (**b**) x = 2, and (**c**) x = 4 AMIs showing brighter FeZr_2_ and darker α-Zr nano-lamellar eutectic microstructure and (**d**) x = 6 and (**e**) x = 10 AMIs showing a heterogeneous type microstructure consisting of mainly α-Zr and Zr_54_Fe_37_Nb_9_ laves phases.

Figure 2a displays the presence of alternate eutectic lamellae of brighter FeZr_2_ and dark α-Zr phases in x = 0 as marked by arrows and have been identified by EDS analysis. A few α-Zr dendrites have been noted to be present along with a eutectic matrix. Similarly, the microstructures of x = 2 and x = 4 samples show a eutectic matrix, as shown in Figure 2b,c, respectively. Table 1 summarizes the constituent phases in different Nb containing composites, as identified using XRD and EDS analyses. A new phase with chemical composition Zr_54_Fe_37_Nb_9_, (atom %) evolved in the x = 4 sample, and the morphology was modified from the lamellar eutectic to a complex heterogeneous microstructure, consisting of mainly α-Zr and Zr_54_Fe_37_Nb_9_, as also observed in x = 6 (Figure 2d) and x = 10 (Figure 2e). The volume fraction (vol %) of the phases present in these samples are shown in Table 1. The amount of α-Zr phase remained constant around 69–74 vol % when the Nb content varied from 0 to 6 atom %. The α-Zr showed a decrease when Nb content increased to 10 vol %. On the other hand, the amount of FeZr_2_ phase decreased from 31 vol % in the x = 0 sample to 12 vol % in the x = 4 sample and finally disappeared when Nb content increased to 6 atom %. The ternary intermetallic phase is not observed in the sample without Nb content. With the addition of Nb (x = 2), the ternary intermetallic phases form and its fraction increased from 8 to 39 vol % when the Nb content increased from 2 to 10 atom %, respectively. Therefore, the addition of Nb helped to destabilize the FeZr_2_ phase and promoted the formation of the Zr_54_Fe_37_Nb_9_ intermetallic compound, as corroborated by the XRD data.

### 3.3. TEM Analysis

**Figure 3:**
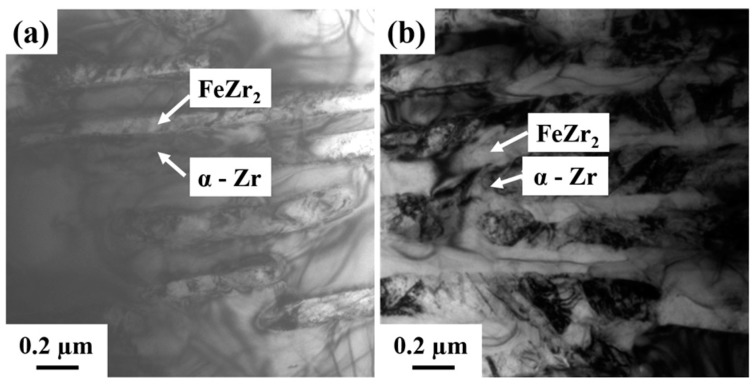
Transmission electron microscopy—bright field images of (**a**) x = 0 and (**b**) x = 2 samples showing the alternate nano-lamellar structures of the α-Zr and FeZr_2_ phases.

Figure 3 shows bright field (BF) TEM images of the x = 0 and x = 2 samples, respectively. Figure 3a shows the alternating two-phase lamellar eutectic structure of the x = 0 sample. The darker lamellae were identified as hcp α-Zr, and the brighter lamellae as tetragonal FeZr_2_, as deduced from SAED patterns. Similarly, a eutectic microstructure is revealed in Figure 3b for the x = 2 sample. The interlamellar spacing (λ = (λ_α-Zr_ + λ_FeZr2_)/2) was determined by measuring the lowest possible *λ* values at 10 different locations. The lowest values of *λ* were measured to be 120 nm, 135 nm, and 300 nm in the x = 0, x = 2, and x = 6 samples, respectively. Therefore, the addition of Nb increases the *λ* value and causes a coarsening of the microstructure.

### 3.4. Compressive Deformation Behavior

**Figure 4:**
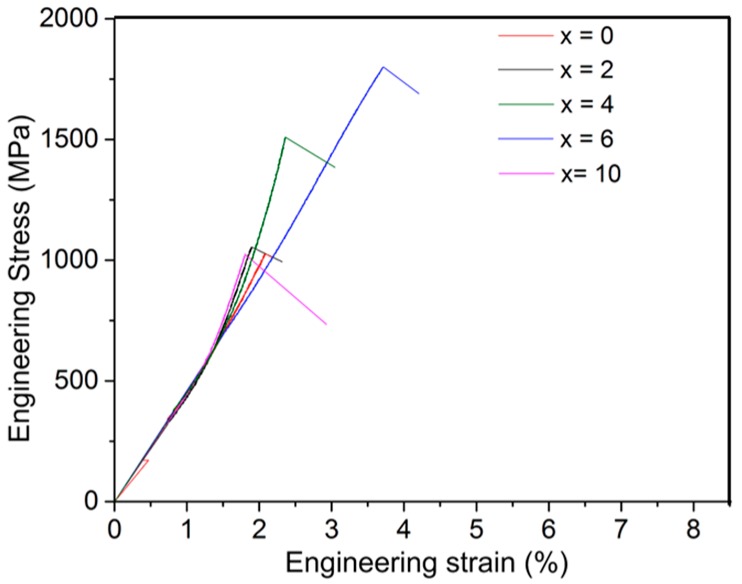
The engineering stress–strain curves of (Zr_0.76_Fe_0.24_)_100−x_Nb_x_ eutectic composites under compression at room temperature.

The compressive engineering stress–strain plots of Zr–Fe–(Nb) NECs as obtained during the uniaxial compression tests (CT) under constrained geometry at room temperature are plotted in Figure 4. The yield strength (*σ_y_*), fracture strength (*σ_f_*), and fracture strain (*ε_f_*) of the investigated NECs are measured and summarized in Table 1. The sample without Nb had a high value of *σ_y_* = 1015 MPa and *σ_f_* = 1025 MPa, with a low fracture strain of *ε_f_* = 2.1%. The stress–strain curves of the investigated NECs show primarily elastic deformation with very limited plastic strain, as evident in Figure 4. Therefore, their *σ_y_* values of all the NECs are close to their *σ_f_* values. The compression test revealed an increase in fracture strength *σ_f_* for the composites with an increase in Nb content. In the x = 2, x = 4 and x = 6 NECs, the measured *σ_f_* was found to be increased up to 1060 MPa, 1510 MPa, and 1800 MPa, respectively. The fracture strain *ε_f_* gradually increased from 2.1% up to 3.7% upon the addition of Nb. A sudden drop in *σ_f_* was observed in the x = 10 NEC, which had a low fracture strength of *σ_f_* = 1025 MPa with a reduced strain of *ε_f_* = 1.8%. The presence of the eutectic microstructure and an increase in the volume fraction of the ternary intermetallic phase improved the mechanical properties of these samples (with increasing addition of Nb). The best mechanical properties were obtained in the x = 6 condition, where there was an optimum mix of α-Zr and Zr_54_Fe_37_Nb_9_ phases. Further increase in the amount of brittle Zr_54_Fe_37_Nb_9_ phase degraded the properties of these (Zr_0.76_Fe_0.24_)_100−x_Nb_x_ composites.

### 3.5. Elastic Modulus Measurement 

The density of Zr–Fe–(Nb) composites were measured by the Archimedes principle and are listed in Table 2. Nb addition increased the density (*ρ*) of the composites from 6.74 g/cc (x = 0) to 7.03 g/cc (x = 10). The estimated Poisson’s ratio *ν*, Young’s modulus *E*, bulk modulus *G*, and shear modulus *K* is summarized in Table 2. The addition of Nb gradually increased the Young’s modulus *E* from 68 (x = 0) to 101 GPa (x = 6), the shear modulus *K* from 25 (x = 0) to 38 GPa (x = 6), and the bulk modulus *K* from 94 (x = 0) to 105 GPa (x = 6), respectively. However, *E* = 96 GPa and *K* = 36 GPa was observed in x = 10. Nb addition increased the density of the composites and caused significant changes in their elastic moduli due to the alteration in the phase constituents and subsequently the microstructure.

### 3.6. Vickers Bulk Hardness

**Figure 5:**
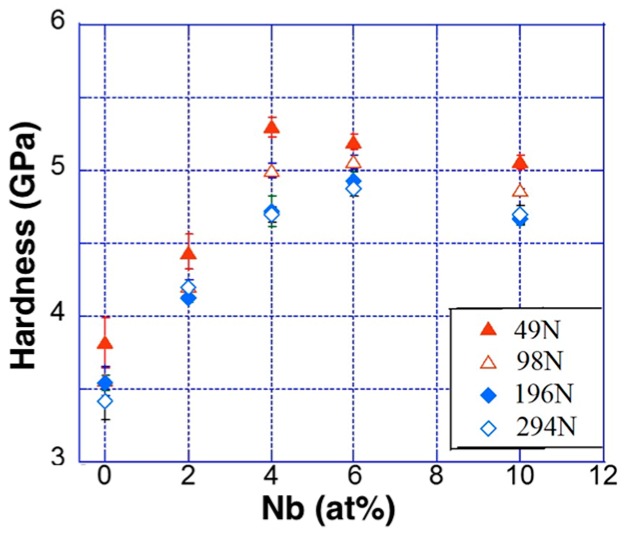
Plots of measured *H* vs. Nb content as a function of loads *P_max_* from 49 N up to 294 N in (Zr_0.76_Fe_0.24_)_100−x_Nb_x_ composites.

The macro-hardness (*H*) data at different load *P_max_* values is summarized in Table 3. Figure 5 shows the variation of *H* with the addition of Nb. A high value of *H* = 3.82 ± 0.17 GPa was obtained in the x = 0 sample at *P_max_* = 49 N. However, *H* further increased up to 5.20 ± 0.05 GPa at *P_max_* = 49 N in x = 6. Thus, *H* gradually increased upon the addition of Nb, which suggests an increase in composite strength. However, a sudden drop in hardness of 5.06 ± 0.05 GPa was observed in the x = 6 sample at *P_max_* = 49 N, and the intermetallic Zr_54_Fe_37_Nb_9_ phase had a big impact on the higher hardness values. The high volume fraction of Zr_54_Fe_37_Nb_9_ was present in the x = 10 sample, as revealed in XRD analysis and SEM micrographs, lowering the *H* values. Therefore, the *H* of the investigated NECs depended on the relative volume fraction of α-Zr, FeZr_2_, and Zr_54_Fe_37_Nb_9_, as shown in Table 3.

### 3.7. Indentation Fracture Toughness

**Figure 6:**
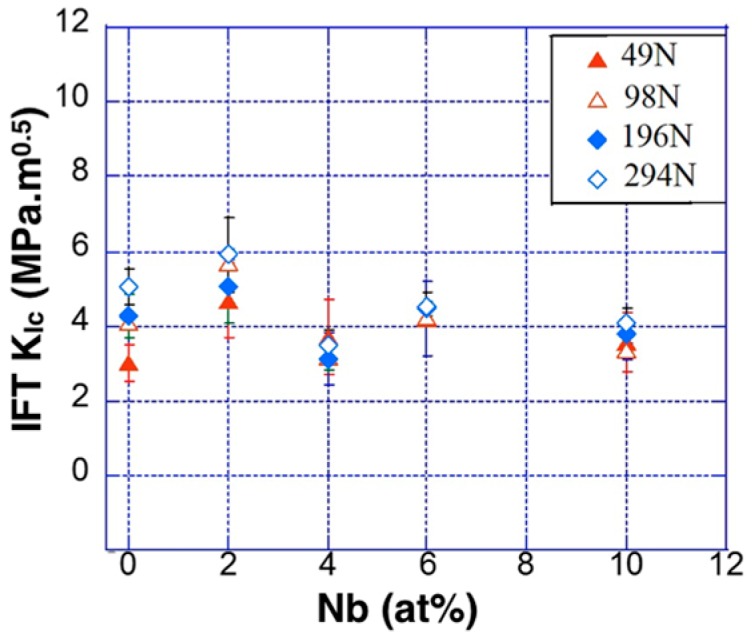
Plots of measured value of *K_IC_* vs. Nb content in (Zr_0.76_Fe_0.24_)_100−x_Nb_x_ composites at different *P_max_* values in the range of 49 N up to 294 N.

The Vickers IFT measurements refer to a complex state of three-dimensional crack system with significant deformation residual stress and damage around the cracks. In this investigation, *l*/*a* data was considered to be in the range of 0.25–2.5. The values of calculated *K_IC_* of the investigated NECs are summarized in Table 3. The length of the cracks extending from the four corners (*l*) and the size of the indentation diagonal (*d*) was measured for the desired Palmqvist crack lengths and the size of indentation with an standard error in the range of 2–10%. The IFT measurements were performed at various *P_max_* values within a range between 49 and 294 N in order to study the effect of load variation and Nb content on the *K_IC_* in Zr–Fe–(Nb) NECs. Figure 6 shows the variation of the estimated *K_IC_* measured at different *P_max_* values. *K_IC_* gradually increased from 3.0 ± 0.5 in the x = 0 sample at *P_max_* = 49 N to 4.2 ± 1 MPa√m in the x = 6 sample at *P_max_* = 49 N; thereafter, it dropped down to 3.6 ± 0.8 MPa√m at x = 10 at *P_max_* = 49 N. In addition, the estimated *K_IC_* gradually increased with the increase in applied indentation load *P_max_*. Such as in case of the x = 0 AMI, *K_IC_* increased from 3.0 ± 0.5 √m at *P_max_* = 49 N up to 5.1 ± 0.5 MPa√m at *P_max_* = 294 N. Therefore, the fracture resistance of the investigated composites increased with the increase in Nb content in the NECs up to x = 2. The evolution of intermetallic Zr_54_Fe_37_Nb_9_ decreased the IFT values. By increasing the Nb up to x = 6 atom %, the fracture strength was enhanced due to the presence of nanolamellar α-Zr and FeZr_2_ phases and solid-solution strengthening. However, the presence of homogeneous NECs (x = 0 and 2) with a higher volume fraction of FeZr_2_ phase led to IFT values higher than the other Nb-containing specimens.

### 3.8. Fractrographic Investigation

**Figure 7:**
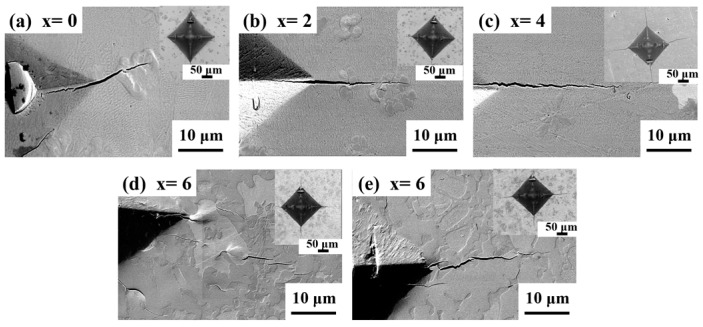
Secondary scanning electron microscopy images of indented impression along with Palmqvist cracks emerged from the edge of the indentation diagonals and crack deflection on the surface in (Zr_0.76_Fe_0.24_)_100−x_Nb_x_. Inset: Scanning electron microscopy images showing the impression of the indentation N, with Nb content varying from (**a**) x = 0, (**b**) x = 2, (**c**) x = 4, (**d**) x = 6 and (**e**) x = 10 respectively.

To understand the IFT behavior of the NECs with the nanolamellar phases, the indented and fractured NECs were further investigated in detail. Figure 7 shows the SEM images of the lateral surfaces of the indented and fractured specimens. Palmqvist cracks were observed emerging from the edges of the indentation impressions. The geometry of Palmqvist cracks at the edges of the indentations at an operating load of 49 N is shown in Figure 7, which shows the SEM micrograph of the indented impression along with the Palmqvist cracks emerged from the edge of the indentation diagonals in the x = 0 and x = 6 NECs. It is interesting to note that the plastic flow lines in the α-Zr phase were observed near the vicinity of the indented plastic zone in the x = 6 samples, while the other phases do not show plastic flow lines. These results suggest that the mutual interaction between lamellar FeZr_2_//α-Zr phases is responsible for an enhanced fracture strength and fracture toughness.

## 4. Summary

A series of eutectic nano-lamellar α-Zr//FeZr_2_ eutectic composites was developed in Zr–Fe–(Nb) at low cooling rates (10 K/s). Nb addition resulted in the formation of intermetallic Zr_54_Fe_37_Nb_9_ and destabilized the FeZr_2_ phase. Even though Nb addition coarsened the interlamellar spacing λ, a compressive fracture strength up to 1800 MPa was achieved. On the other hand, Nb addition increased the density, the Young’s modulus, and the hardness of the composite. The fracture resistance of the NECs increased with the increase in Nb from 3.1 ± 0.5 (x = 0) up to 4.7 ± 1.0 MPa√m (x = 2) at *P_max_* = 49 N. These results suggest that the mutual interaction between nanolamellar FeZr_2_//α-Zr phases is responsible for an enhanced IFT and a high fracture strength, whereas a higher volume fraction of Zr_54_Fe_37_Nb_9_ intermetallic phase in x = 10 is responsible for reduced IFT values.

## Figures and Tables

**Table 1:** Phase constituents (including their volume fraction) and the corresponding mechanical properties of (Zr_0.76_Fe_0.24_)_100−x_Nb_x_ composites.

Alloys	Phases	α-Zr	FeZr_2_	Zr_54_Fe_37_Nb_9_	*σ_y_* (MPa)	*σ_f_* (MPa)	*ε_f_* (%)
x = 0	α-Zr + FeZr_2_	69	31	-	1015	1025	2.1
x = 2	α-Zr + Zr_54_Fe_37_Nb_9_ + FeZr_2_	74	18	8	1055	1060	2.0
x = 4	α-Zr + Zr_54_Fe_37_Nb_9_ + FeZr_2_	72	12	16	1510	1510	2.4
x = 6	Zr_54_Fe_37_Nb_9_ + α-Zr	72	-	28	1800	1800	3.7
x = 10	Zr_54_Fe_37_Nb_9_ + α-Zr	61	-	39	1025	1025	1.8

**Table 2:** The density (*ρ*), Poisson’s ratio (*ν*), Young’s modulus (*E*), bulk modulus (*K*), and shear modulus (*G*) of (Zr_0.76_Fe_0.24_)_100−x_Nb_x_ composites.

Alloys	*ρ* (g/cc)	*ν*	*E* (GPa)	*K* (GPa)	*G* (GPa)
x = 0	6.74	0.3787	68	25	94
x = 2	6.82	0.3540	87	32	99
x = 4	6.88	0.3396	97	36	101
x = 6	6.90	0.3396	101	38	105
x = 10	7.03	0.3499	96	36	107

**Table 3:** Vickers bulk hardness (*H*), indentation fracture toughness (*K_IC_*), and Palmqvist crack length (*l*) at different applied *P_max_* values.

Alloys	*P_max_* (N)	*H* (GPa)	*l* (µm)	*K_IC_* (MPa√m)
x = 0	49	3.82 ± 0.17	33.09 ± 10	3.02 ± 0.5
	98	3.56 ± 0.10	33.68 ± 5	4.08 ± 0.2
	196	3.54 ± 0.05	64.22 ± 16	4.27 ± 0.6
	294	3.42 ± 0.13	67.43 ± 17	5.05 ± 0.5
x = 2	49	4.44 ± 0.12	18.69 ± 9	4.67 ± 1.0
	98	4.21 ± 0.04	21.70 ± 1	5.70 ± 0.1
	196	4.13 ± 0.04	59.48 ± 19	5.07 ± 1.0
	294	4.20 ± 0.02	65.26 ± 23	5.90 ± 1.0
x = 4	49	5.30 ± 0.07	34.08 ± 14	3.71 ± 1.0
	98	5.00 ± 0.05	87.63 ± 22	3.15 ± 0.7
	196	4.72 ± 0.10	166.27 ± 30	3.12 ± 0.3
	294	4.70 ± 0.05	201.66 ± 50	3.50 ± 0.4
x = 6	49	5.20 ± 0.05	26.38 ± 9	4.18 ± 1.0
	98	5.06 ± 0.05	52.55 ± 17	4.21 ± 1.0
	196	4.93 ± 0.06	82.73 ± 8	4.48 ± 0.2
	294	4.88 ± 0.06	122.4 ± 18	4.52 ± 0.4
x = 10	49	5.06 ± 0.05	34.47 ± 3	3.56 ± 0.8
	98	4.87 ± 0.01	72.60 ± 3	3.33 ± 0.2
	196	4.67 ± 0.04	112.5 ± 4	3.78 ± 0.4
	294	4.70 ± 0.06	144.9 ± 4	4.08 ± 0.4
